# Residential and inpatient treatment of substance use disorders in Sub-Saharan Africa: a scoping review

**DOI:** 10.1186/s13011-023-00589-0

**Published:** 2024-01-11

**Authors:** Samuel Janson, Lily Nyenga, Haneefa Saleem, Larissa Jennings Mayo-Wilson, Stella E. Mushy, Masunga K. Iseselo, Jenna van Draanen, Joseph Tucker, Mecca McPherson, Donaldson F. Conserve

**Affiliations:** 1 The George Washington University Milken Institute School of Public Health, Washington, DC USA; 2 The Johns Hopkins University Bloomberg School of Public Health, Baltimore, MD USA; 3 The University of North Carolina Gillings School of Public Health, Chapel Hill, North Carolina USA; 4 Muhimbili University of Health and Allied Sciences, Dar Es Salaam, Tanzania; 5 University of Washington School of Public Health, Seattle, Washington USA; 6 University of Washington School of Nursing, Seattle, Washington USA; 7 University of North Carolina School of Medicine, Chapell Hill, North Carolina USA; 8 Temple University College of Public Health, Philadelphia, PA USA

**Keywords:** Substance use disorders, Sub-Saharan Africa, Addiction, Drug and alcohol treatment, Drug, And alcohol rehabilitation

## Abstract

**Background:**

With substance use rates increasing in Sub-Saharan Africa (SSA), an understanding of the accessibility and effectiveness of rehabilitative services for people who use alcohol and other drugs (AOD) is critical in the global efforts to diagnose and treat substance use disorders (SUD). This scoping review seeks to address the gaps in knowledge related to the types of research that have been conducted regarding inpatient or residential SUD treatment in SSA, the settings in which the research was conducted, and the study countries.

**Methods:**

A search of three databases, PubMED, Scopus, and African Index Medicus, was conducted for publications related to the treatment of SUD in inpatient or residential settings in SSA. Articles were screened at the title/abstract level and at full text by two reviewers. Articles eligible for inclusion were original research, conducted in SSA, published in English, included populations who received or were currently receiving treatment for SUD in inpatient or residential settings, or documented demand for SUD services.

**Results:**

This scoping review included 82 studies originating from 6 countries in SSA. Three themes emerged within the literature: access and demand for inpatient and residential SUD treatment, quality and outcomes of SUD treatment, and descriptions of the services offered and staffing of these facilities. Barriers to access include financial barriers, limited availability of services, and geographic concentration in cities. Women were shown to access residential and inpatient SUD treatment at lower rates than men, and certain racial groups face unique language and financial barriers in accessing services. Studies indicate mixed success of inpatient and residential SUD treatment in sustained SUD remission for patients.

**Conclusion:**

There are significant gaps in the literature, driven by a lack of longitudinal studies focused on patient outcomes following treatment and the use of a narrow definition of treatment success. Both structural and non-structural barriers, such as stigma and discrimination, are barriers to access. Further research is needed to evaluate approaches to mitigate these barriers and expand access to residential and inpatient SUD treatment.

**Supplementary Information:**

The online version contains supplementary material available at 10.1186/s13011-023-00589-0.

## Introduction

Across the world, rates of substance use are rising. In 2021 alone, 1 in 17 people (aged 15–64) globally used illicit drugs, representing a 23% increase from the preceding decade [1]. Among those who used drugs in 2021, 39.5 million people were estimated to meet the criteria for a drug use disorder [1]. The increases in drug use throughout the world are not driven across age groups equally. For example, 70% of those who sought drug treatment in 2021 in Africa were under 35 years old [1]. The demand for drugs across the African continent is expected to continue to rise, with some projections predicting a 150% increase in illicit drug consumption by 2050, representing 14 million new people using drugs [2]. The global issue of harmful substance use does not only involve illicit drugs. Alcohol is the cause of 5.9% of all deaths globally, representing 3 million deaths per year [3]. Similar to other drugs, alcohol has been shown to affect young adults at a disproportionate rate, accounting for 25% of the deaths of people aged 20–29 [3]. In Africa, alcohol use was attributed to 6.4% of deaths on the continent in 2012 and is the leading risk factor for Disability Adjusted Life Years (DALYs) among adolescents and young adults (aged 15–24) and [4, 5].

The African Union (AU) has cited substance use as a primary challenge in achieving the United Nations Sustainable Development Goals (SDGs) as well as the AU Agenda 2063. In the African Union’s (AU) *Plan of Action on Drug Control and Crime (2019–2023),* member states from the five regions of the African continent all reported rising rates of illicit drug consumption in 2018 [6]. This problem is driven by increased demand for illicit drugs among African people, as well as the increased presence of drug production sites throughout the continent [6]. While the African Union has developed action plans to address this issue since 1996 [6], substance use disorders (SUD) and associated diseases have continued to pose a substantial challenge to the public health and economy of Africa.

## Rationale

Critical to combatting rising rates of SUD throughout the world, especially in Sub-Saharan Africa (SSA), is a comprehensive understanding of the treatment options that are available to people with SUD. However, formal treatment should be understood as just one path that individuals take to initiate and sustain recovery, in addition to the various peer-supported or solo approaches that are utilized [7]. While there are multiple definitions of recovery employed throughout the world, the Betty Ford Institute’s Consensus Panel posits that *sobriety*, defined as abstinence from alcohol and nonprescribed drugs, is just one component of a three-part definition of recovery [8]. The other components include *personal health*, defined as a holistic state of well-being rather than just a reduction in symptoms, as well as *citizenship*, which includes service to one’s community [8]. This broader definition is important in the exploration of the SUD treatment literature, particularly as it relates to conceptualizations of treatment success or failure.

Globally, the rates of treatment for individuals with SUD are low, and the estimates of treatment access decrease with the economic status of the country, ranging from high-income (10.3%) and upper-middle income (4.3%) to low and lower-middle income (1%) [9]. The World Health Organization (WHO) and the United Nations Office on Drugs and Crime (UNODC) have put forth standards for drug treatment including that treatment should be available, accessible, affordable, evidence-based, and diversified [10]. Part of this diversification includes a variety of treatment-delivery settings including community outreach, inpatient, outpatient, and residential venues [10]. Short-term inpatient and long-term residential treatment venues have been identified as generally appropriate for people with more complex or chronic SUD, including those for whom outpatient treatment has had a lower treatment effect [10].

This scoping review aims to explore the literature on inpatient and residential treatment of SUD, including both drugs and alcohol, in SSA. The focus of this review on inpatient and residential treatment of substance use was due to the study team’s identification of another scoping review that focused on prevention efforts [11], but failure to identify a review that specifically focused on the inpatient or residential treatment literature. Due to the resource-intensive nature of inpatient and residential SUD treatment options and the growing demand for SUD treatment in SSA, this scoping review sought to assess the body of literature on this topic guided by three research questions: (1) In what settings and which countries have studies been conducted? (2) What types of studies have been conducted? (3) Where are the gaps in the literature related to inpatient or residential SUD treatment in SSA?

## Methods

### Design

This scoping review was designed in accordance with the guidelines of Preferred Reporting Items for Systematic Reviews and Meta-Analyses extension for Scoping Reviews (PRISMA-ScR) [12].

### Search strategy

A search strategy was developed in collaboration with a research librarian from the George Washington University School of Medicine. Three databases were included in the search: PubMED, Scopus, and African Index Medicus. All searches were conducted on April 10, 2023. Search terms are included in Additional file 1.

### Inclusion criteria

To be eligible for inclusion, studies had to be: original research, based in SSA, published in peer-reviewed journals, written in the English language, involve data collected from patients being treated for SUD in inpatient/residential settings, staff working in inpatient/residential settings, or data from those who are not in treatment if the study was focused on barriers to accessing residential/inpatient SUD care.

“Inpatient” was defined as a clinical setting where patients receive SUD treatment such as a health center or hospital, and where patients are kept overnight for either a brief or extended period of time during the duration of their SUD treatment. “Residential” was defined as a non-clinical setting, such as a halfway house, sober house, drug rehabilitation facility outside of a hospital, or other treatment setting where the patient is staying overnight in the treatment facility or a residential venue that is overseen by the treatment provider. These venues are contrasted with outpatient and community settings, where patients receive SUD treatment services but are not staying overnight in the treatment facility. Treatment was defined as any clinical or non-clinical activity with the primary objective of helping a patient to reduce or cease substance use, including but not limited to detoxification, group meetings, pharmacotherapy, individual counseling, general health services for people in SUD treatment, pro-social activities, and SUD-focused psychoeducation.

In the original search, studies were not limited to any specific time period. However, a later inclusion criterion was added to only include studies published from 2000 to the date of the search. This decision was due to initial reviews of the older literature which indicated that these studies did not provide an accurate picture of the contemporary state of SUD treatment in SSA. The researchers had a stronger focus on contemporary SUD treatment research, and for this reason, decided to add this additional criterion.

### Exclusion criteria

Excluded from the study were editorials, commentaries, conference abstracts, grey literature, meta-analyses, literature reviews (including systematic and scoping reviews), as well as any publication that did not meet the inclusion criteria.

### Selection of studies

**Fig. 1:**
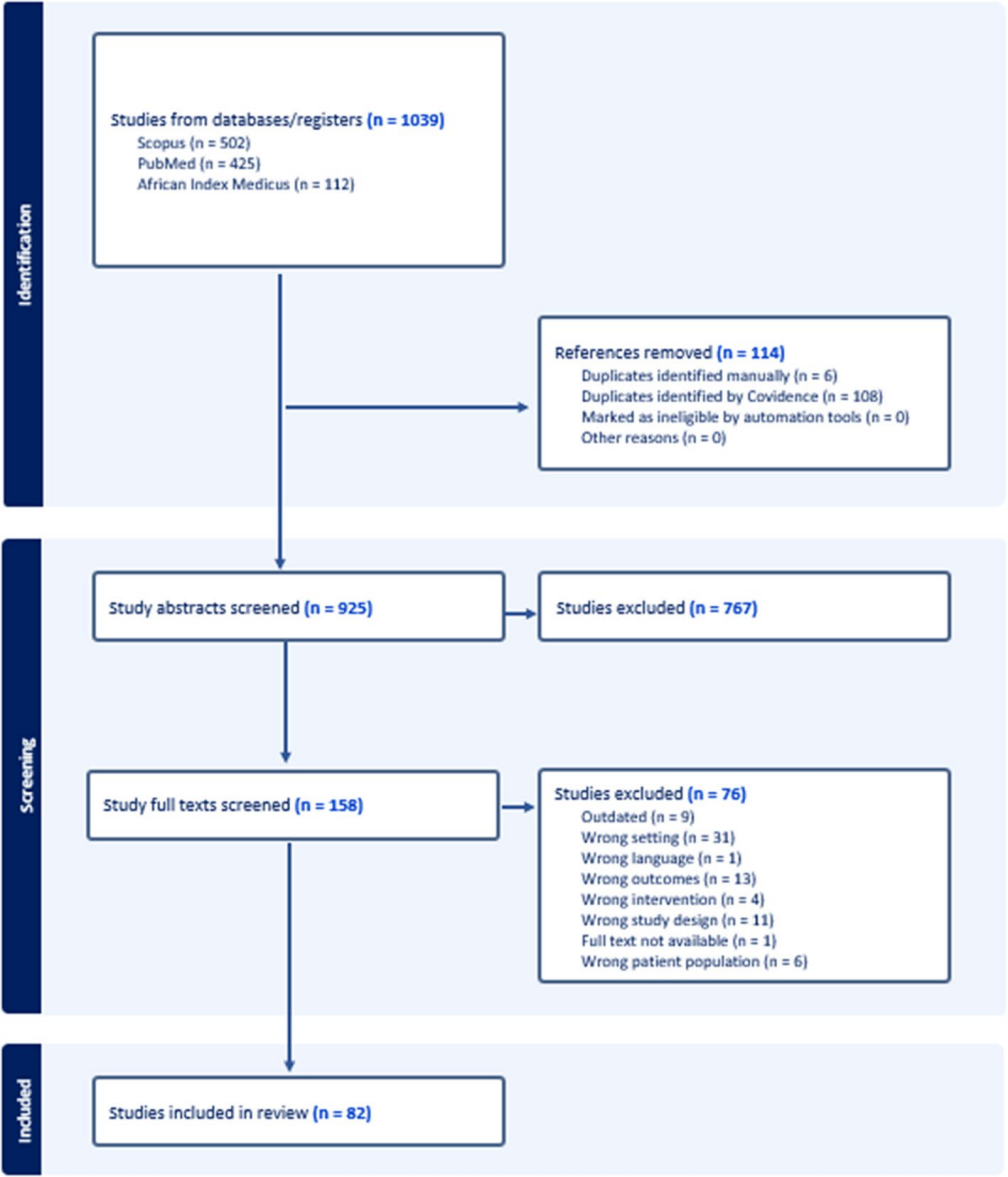
PRISMA flow chart

Following the search, two reviewers, SJ (MPH) and LN (MPH), conducted a multi-step screening process of the publications using the Covidence web-based collaboration software platform that streamlines the production of systematic and other literature reviews. First, studies underwent a title and abstract screening where publications were independently evaluated by each reviewer who voted on whether to include the study in a full-text screening. Following this step, the two reviewers met to discuss conflicts. If a consensus could not be reached based on the title and abstract, the article defaulted to a full-text review where a more thorough evaluation of the study could be completed. After this step, the two reviewers conducted a full-text review. The reviewers met again after the full-text screening to discuss conflicts until a consensus was reached. If a consensus could not be reached, the final decision was made by co-author DFC (PhD). Publications that were agreed upon by both reviewers as meeting the inclusion criteria were included in the review. The process is outlined below in Fig. 1.

### Data extraction

Articles that were found to be eligible for inclusion after a full-text screening were logged in a Microsoft Excel file that included: the study title, author, study country, year of publication, the country affiliation of the first author, and key results of the study.

## Results and synthesis

### Search results

The search yielded 1,039 articles, of which 114 were identified as duplicates. Through title and abstract screening, 767 studies did not meet the eligibility criteria, which left 158 for full-text screening. Following full-text screening, 82 studies were found to be eligible for inclusion in the scoping review. The most common reason for excluding studies was that the setting did not meet the inclusion criteria, indicating that the studies likely took place in an outpatient or community-based venue. A flow chart documenting the screening process can be found below.

### Characteristics of included studies

While studies from all SSA countries were eligible for inclusion, publications included in the review came from just six countries. The overwhelming majority of studies were from South Africa which represented 63 studies, followed by Kenya (7), Nigeria (5), Uganda (4), Ghana (2), and The Gambia (1). The most common first author affiliation was also South Africa (54), followed by The United States (7), Kenya (6), Nigeria (5), Ghana (2), Germany (2), Uganda (2), The Gambia (1), United Kingdom (1), Switzerland (1), and Zimbabwe (1).

The studies included in the review encompassed a range of study designs and research methodologies including: cross-sectional studies (51 studies), cohort (8 studies), case studies (4 studies), case–control (2), and qualitative (17). The results of the scoping review indicate that research concerning inpatient and residential SUD treatment accelerated after 2010, with 17 included articles published before 2010 and 68 between 2010 and 2023.

### Synthesis of results

Following the conclusion of the full-text screening, reviewers summarized the findings of each of the studies (Additional file 2). Each reviewer then performed a qualitative analysis of the summaries and proposed broad themes for grouping the studies. From this process, three major themes emerged: demand for treatment and access to treatment, the utilization of quality and outcomes measures of patients who receive SUD treatment in inpatient/residential settings, and descriptions of treatment services and staffing of SUD treatment facilities. The key aspects of these themes are included below in Table 1. Following this step, the reviewers then began to formulate sub-themes that emerged within the literature. Sub-themes were established to achieve conceptual saturation of all the topics presented in the literature. A Microsoft Excel spreadsheet was created with all the study titles and sub-themes on different axes and the corresponding cells were populated with relevant data extracted from each of the studies.

**Table 1:** Keys aspects of the three themes

Theme	Key Aspects
Treatment Demand and Access	• The existence of monitoring systems to collect information relating patients in SUD treatment over time • The intersection of SUD with psychiatric needs including the treatment of people with cooccurring psychiatric illness and SUD in psychiatric facilities • The role of COVID-19 in treatment-seeking and access • Factors that hinder treatment access for historically disadvantaged groups including women and certain racial and ethnic groups • Financial barriers to treatment including out-of-pocket payments, lack of insurance coverage, and a lack of insurance-accepting facilities • Uneven geographic distribution of SUD services
Quality and Outcomes	• The presence of systems for monitoring service quality and patient outcomes, and barriers and facilitators to implementation of these systems in treatment centers • Definitions of treatment success and presentation of patient outcomes data
Description of Services, Staffing, and Treatment Models	• Services offered in facilities including mental health counseling, provision of psychiatric and SUD medication, group therapy, etc • Treatment philosophies/approaches used in residential or inpatient facilities • Staffing of inpatient and residential facilities including the education and professional credentials of staff, staff size, and age/race/language demographics of SUD workforce

## Treatment demand and access

### Primary substances of treatment-seekers

The availability of surveillance data related to SUD treatment demand and uptake varied by country, with South Africa and Nigeria benefiting from the presence of a coordinated national surveillance system that monitors treatment centers for trends in substance use demand and the demographics of individuals accessing services [13, 14]. These surveillance systems were started in 1996 [13] and 2015 [14], respectively.

The literature indicates that the types of substances driving treatment demand have changed from the beginning of the 21st century to the present day. In South Africa, studies show that the proportion of treatment-seekers whose primary substance was alcohol began to decline in the early 2000s, as cannabis and “white pipe” (a combination of cannabis and Mandrax) were increasingly cited as the primary drugs of choice by those in treatment [15]. In more recent publications based in the Gauteng and Western Cape Provinces, heroin and methamphetamine were the primary substances of the two sample populations [16, 17]. In Nigeria, treatment center surveillance compared patients in treatment in the early 2000s to those a decade previously and found that a greater proportion of those in treatment were citing cannabis as their primary substance, compared to cocaine in the previous sample [18]. Additionally, data from 2015–2018 taken from Nigeria show rising rates of opioid usage among treatment-seekers, as well as persistently high rates of cannabis use [19].

In other countries, the data regarding the primary substances of use were more limited. One study from Uganda across 10 treatment facilities found that the most commonly used substance was alcohol, followed by cannabis and cocaine [20]. A study from the Gambia found that cannabis was the most commonly used substance among treatment-seekers, often used in combination with stimulants or tranquilizers [21].

### The intersection of SUD and psychiatric treatment needs

The intersection of the demand and treatment for both SUD and psychiatric medical care was documented in the literature. Across countries, psychiatric facilities were shown to be utilized for the treatment of SUD for patients with and without additional psychiatric diagnoses. In Kenya, three studies from psychiatric facilities documented rates of SUD among the sample populations, reporting rates between 7% and 34.4% [22–24]. In a study from a psychiatric hospital in the Gambia, more than one-third of the sample population (35%) met the diagnostic criteria for a psychiatric or mood disorder in addition to SUD [21]. Many of the studies included in this review from Nigeria were conducted in a neuropsychiatric facility [18, 19, 25, 26], and researchers noted that the location of SUD treatment in psychiatric facilities may contribute to low uptake of SUD services [26].

Rates of substance use among patients admitted to psychiatric hospitals in South Africa were shown to be high, ranging between 40 and 79% [27–31]. In studies that reported the primary substance of use among patients in psychiatric units, cannabis, methamphetamine, and alcohol were the most often reported [30, 31]. Heavy substance use among psychiatric patients was reported to complicate the provision of services in psychiatric hospitals, as healthcare workers reported an increased burden on staff when treating people who use methamphetamine [31].

### The impact of COVID-19 on service provision and utilization

In more recent publications, the impact of COVID-19 on SUD treatment availability and utilization emerged as a research topic. From South Africa, one study that examined the impact of COVID-19 on hospital admissions found that while total hospital admissions declined during the pandemic, hospitalizations for acute alcohol withdrawal increased, likely the result of a ban on alcohol sales during the national lockdown [32]. An additional study of SUD service providers found that most service providers felt that while demand had remained constant during the COVID-19 pandemic, the availability of services declined as many facilities limited their patient capacity [33].

### Barriers to access for women and certain racial groups

Across countries, studies indicated the underrepresentation of women and certain racial groups in inpatient or residential SUD treatment. Analysis of the literature indicates that men in Kenya likely access residential and inpatient SUD services at higher rates than women, as men were a greater proportion of participants in all of the sample populations in studies from Kenya that reported the demographic information [22, 24, 34]. In Nigeria, one study noted the overrepresentation of men in the sample population, who accounted for over 90% of patients in treatment while over a quarter of people who use drugs (PWUD) in Nigeria are women [26].

A case–control study from South Africa comparing PWUD in treatment to those who are not in treatment found that women and Black South Africans are underrepresented in SUD treatment [35]. The reasons posited for the gender disparity include greater instances of stigma towards women who use drugs, fear among women that they will lose their children if they present for treatment, and discrimination in healthcare settings [35, 36]. Studies also indicate that many of the same barriers that prevent men from receiving SUD treatment such as financial constraints, lack of transportation, and low awareness of treatment options, also hinder women’s access but at a greater intensity [36]. Some of the proposed solutions to the underrepresentation of women include the better integration of SUD treatment with primary care and sexual health services and stigma reduction strategies [37, 38].

Related to barriers to access for Black South Africans, one barrier that emerged is the languages spoken in treatment facilities [38, 39]. Residential SUD treatment providers were shown to be disproportionately white (36.4%) and to speak Afrikaans (36.6%) or English (33.8%) as their first language [39]. Outpatient facilities were found to be more likely to offer services in Indigenous African languages, as well as to perform specific outreach activities to communities underrepresented in SUD treatment, and thus were more effective in the promotion of their services to Black South Africans [40]. Additionally, the literature highlighted the disproportionate impact that financial barriers have on Black South Africans [40]. While there may be some limited financial assistance for SUD treatment, particularly in government-run facilities, practical barriers such as a lack of money for transportation still inhibit treatment access for Black South Africans [40].

### Financial barriers to treatment access

Financial barriers to accessing treatment were commonly cited throughout the literature. From a cross-sectional study in Kenya of 6 treatment facilities, only one of these facilities accepted insurance, and the out-of-pocket cost for a 90-day stay ranged between 700 and 2,000 USD [41]. A heavy reliance on out-of-pocket payments was also observed in a psychiatric hospital treating patients with SUD, where more than 70% of patients paid out of pocket [23]. The cost barrier to treatment was echoed by a study of people who use heroin in three Kenyan cities, who reported a strong desire to receive residential treatment but inability due to an average cost of 114 USD per month [42]. Financial barriers were also shown to hinder treatment access in South Africa [41, 43].

In Uganda, the cost of residential treatment was shown to impact the patient population accessing treatment, as well as the service offerings of the facilities. One high-cost facility in Kampala was reported to cost 20 USD per day, with most residents staying at least three months [44]. The individuals receiving treatment in this facility were generally from wealthy families, and many had either lived or traveled abroad. This facility was contrasted with outreach to poorer people with SUD in the Kampala area, which consisted more of outpatient mobile services given the reduced operating costs. The non-profit residential program included in the study offered significantly shorter stays for patients, averaging just one week [44].

### Limited availability of services

The literature indicates that countries in SSA often have insufficient capacity within and among residential or inpatient treatment settings to meet the demand for services. In Nigeria, A 2011 nationwide cross-sectional study of 31 treatment facilities found that there were just 566 residential beds dedicated to SUD treatment across 16 residential facilities. Most of the facilities in the sample were run by non-governmental organizations (NGOs) and were heavily reliant on donations, as there was minimal reimbursement available through national insurance schemes [45]. The authors noted that the limited availability of treatment was largely due to a lack of government funding for the building of new facilities, as well as underfunding of existing treatment centers [45].

Data from a survey of treatment centers in Uasin Gishu County, Kenya also indicates a limited bed capacity, where the authors found just 16 beds per 100,000 people in this county [42]. The authors noted that there were no beds dedicated to children or adolescents, and only one-third of beds were allocated for women needing SUD treatment. The authors call for further government investment to address the low density of SUD services available for residents in this county [42]. This call for further government investment in services was echoed in the literature from South Africa, particularly as a remedy for groups that are underrepresented in treatment settings [41, 43].

## Quality and outcomes

### Quality and outcomes monitoring systems

Studies included in this review also explored treatment facilities’ utilization of systems to monitor service quality and patient outcomes. A cross-sectional survey of 55 treatment centers across three provinces in South Africa found that in some areas as many as two-thirds of treatment facilities were not routinely monitoring client treatment outcomes [46]. SUD service providers expressed that while there was a demand for enhanced monitoring and evaluation of program quality and patient outcomes, there were significant barriers to the implementation of a comprehensive evaluation system [47]. These barriers included a lack of computers and the increased time burden the implementation of this system placed on providers [47].

In South Africa in 2008, the Service Quality Measures (SQM) initiative was launched, the first SUD service performance measurement system piloted in a low- or middle-income country [48]. Implementation of the SQM initiative began in 2014 among 10 treatment facilities (residential and outpatient). A 2019 evaluation of the implementation of the SQM initiative showed that overall implementation was high, although there was variability across sites [48]. Service providers indicated three primary drivers related to the degree of implementation: perceived usefulness of the initiative, compatibility with current operations, and simplicity of the intervention [49].

In Nigeria, one cross-sectional study of both residential and non-residential treatment centers found that over half of the facilities in the study did not participate in any form of process or outcomes evaluation [45]. This dearth of evaluation evidence hinders quality improvement efforts and complicates the ability to effectively respond to changing patient needs in SUD treatment. The authors noted that the lack of evaluation measures by these facilities is non-compliant with best practices in SUD treatment [45].

### Patient experiences and outcomes

A prominent theme in the literature was patients’ experiences in treatment, as well as their long-term SUD outcomes after exiting treatment. One commonly assessed patient outcome is treatment completion, defined as whether a patient completes the treatment plan created for them at their respective facility. Two studies from the Western Cape Province reported treatment completion rates of 69% and 59% of their sample populations, respectively [50, 51]. In one of these studies, the presence of a strong therapeutic treatment alliance between patients and providers was found to be the most powerful predictor of treatment completion [50]. In the other study, factors positively correlated with treatment completion included receiving residential treatment rather than outpatient treatment, being older, and having more severe substance use [51].

One study based in Ghana explored patient experiences in treatment and included participants who reported previously being treated for SUD. Participants reported that effective treatment requires service providers to identify patients’ unique sense of purpose in life and suggested that patients’ religiosity should be engaged further to increase treatment efficacy [52]. Additionally, participants noted that families of PWUD in treatment should receive education about supporting their family members in preventing the recurrence of substance use [52].

The success of residential and inpatient SUD treatment in helping patients achieve long-term SUD remission appears variable, with many studies indicating that recurrence of substance use following treatment is common. One prospective cohort study was conducted in South Africa among 300 people who were treated for heroin use disorder in state-funded inpatient facility, which included detoxification and psychosocial support services but did not include the provision of opioid-agonist therapy. At 3-month follow-up, only 6.3% of patients were completely abstinent from substances, though there were significant reductions in patients reporting heroin use (66.5% vs. 100% upon treatment admission) [53]. Treatment stays were on average longer among those who did not report continued heroin use at follow-up (44 days vs. 32 days), but notably, only 11.9% of study participants reported receiving any ongoing formal psychosocial treatment in the community after exiting inpatient treatment [53]. Another study from a residential treatment center in Kampala reported that 65% of discharged patients self-reported remaining drug and alcohol-free one year after treatment completion [44]. This was contrasted with shorter-term SUD treatment programs, where observational outcomes indicate even lower success rates in staying drug and alcohol-free [44]. Additional data taken from 10 treatment facilities in Kampala reported that readmissions into treatment following a return to drug use are common, with 38% of the study population reporting previous SUD treatment [20]. In this sample, repeat treatment episodes were associated with being male, receiving care in private facilities, and being self-employed [20].

## Description of services, staffing, and treatment models

### Services offered and treatment models utilized in facilities

Many studies included in the review indicate similarities between service offerings across countries. In one Ugandan treatment facility, patients undergo medical detoxification and receive medications to manage withdrawals from drugs or alcohol but the study did not give a detailed account of which medications and how they are administered. This program follows the Minnesota Model and reports utilizing various therapeutic sessions including family therapy, occupational therapy, bibliotherapy, group therapy, psychoeducation, and initiation into the principles of Alcoholics Anonymous and Narcotics Anonymous (AA/NA) [44]. Literature from South Africa reported the use of the Minnesota Model as well as the Therapeutic Community (TC) model [54]. South African SUD counselors mentioned most frequently using Cognitive Behavioral Therapy and the Relapse Prevention approaches to therapy and, to a lesser extent, Rogerian (Person-Centered), Solution Focused, and Family Systems therapeutic modalities [39].

The greatest variations across facilities appeared to be the degree to which general medical and psychiatric care is integrated into the SUD treatment facility. One study of residential facilities in Nigeria reported that psychiatric care was provided in 62.5% of facilities, and 75% provided primary care in addition to psychosocial SUD treatment services [45]. From a survey of South African facilities, 56% of facilities offered psychiatric assessments, 73% offered mental health counseling, and 56% offered the provision of psychiatric medications [55]. This study also noted important differences across treatment settings, with inpatient facilities significantly more likely to offer mental health counseling and psychiatric medications [55]. A study from Ghana reported that before reception into the treatment facility, most clients are taken to a psychiatrist for an examination. When this has not occurred, psychiatrists will sometimes visit the facility, particularly to oversee the detoxification of a patient [56], whereas in-house detoxification was shown to be less common in one South African study [55].

Aftercare was another service discussed in the literature. Aftercare typically includes services offered to patients after treatment completion that involves periodic return visits to the treatment facility, and ongoing access to some outpatient services. Aftercare was mentioned as being offered in facilities in Ghana [56], South Africa [55], and Nigeria [45].

There were limited discussions from the literature on the provision of medications for the treatment of SUD (e.g., medication for opioid or alcohol use disorder) in residential or inpatient settings. When medication-assisted therapies were mentioned, it was largely to acknowledge that while these could be beneficial to patients, they were not being used in those facilities [45, 53, 54]. Reasons given for not providing medication for SUD include a lack of country approval, lack of funding, or lack of knowledge among providers of how to prescribe these types of medications [45, 53, 54].

### Staffing

Information relating to the staffing of treatment facilities was also a topic explored in the literature and was closely related to the services offered within a facility. In South Africa, one study reported that 75% of counselors working in SUD treatment were women and that they were ethnically diverse with 36.4% White, 30.8% Black, and 18.9% Coloured (mixed race)^1^ [39]. In terms of education, almost two-thirds (62.3%) of those working in residential rehabilitation had a bachelor’s degree [39]. This sample also found that inpatient settings were more likely to have staff with a graduate degree, compared to outpatient settings [39].

Studies from Nigeria revealed that facilities utilize both full and part-time staff, as well as volunteers [55]. The volunteers assisted the paid staff across a range of clinical and administrative functions, though it was not clear if peer volunteers, those who are also in recovery from SUD, were being utilized in any of the settings. Nurses were reported to be the most common type of staff working in SUD inpatient treatment in Nigeria [55]. In Ghana, the majority of the facilities reported either having a resident or visiting psychologist who provides ongoing psychotherapy to patients [56].

## Discussion

Analysis of the results from this scoping review indicates a few key trends within the literature, as well as some significant gaps. On the first theme, treatment demand and access, both South Africa and Nigeria benefit from having an established SUD treatment surveillance system [13, 14]. Across the countries included in the review, cost appeared to be the most prominent barrier to care, with a heavy reliance on out-of-pocket payment for residential or inpatient SUD treatment [20, 23, 38, 40, 45]. The literature indicates that this cost barrier, particularly in South Africa, has a racialized and gendered effect in terms of who can access SUD treatment services [36–38]. This can be explained further by the fact that South Africa has the highest wealth disparity in the world, which is particularly strong along racial lines, with more than 70% of Black South Africans living in poverty, compared to just 4% of White South Africans [57]. As the South African health system relies heavily on private facilities and providers, many of the more than 80% of uninsured South Africans access healthcare through underfunded and understaffed public facilities [58, 59].

Two other barriers to care that emerged in the literature were gender, with women consistently accessing SUD treatment at lower rates [22–24, 26, 34, 36–39] and linguistic and ethnic discordance between treatment-seekers and providers [38, 39]. A general lack of availability of services was attributed to multiple factors, including a paucity of investment on the part of regional and national governments [40, 42, 43, 45]. This review also found that across countries psychiatric facilities are treating a large volume of patients with SUD, or those with SUD and a cooccurring psychiatric illness [18, 19, 21, 23–31]. Understanding the ability of psychiatric facilities to meet this need, and the appropriateness of these venues for SUD treatment, was a notable gap in the literature. Barriers to access resulting from the COVID-19 pandemic were present in only two studies, but COVID-19 was established as a sub-theme in accordance with the principle of conceptual saturation [60].

On the second theme, quality and outcomes of treatment, South Africa also provided the largest share of information, which was assisted by a coordinated evaluation and quality improvement measurement system nationally, the SQM initiative [49–51]. Across countries, there was a noted demand for more quality and outcomes monitoring, but logistical challenges including financing, technological barriers, and a lack of knowledge were identified as barriers to further adoption [46, 49–51] One major threat to high-quality service and long-term SUD remission for patients is failure to complete the full course of their treatment [50, 51]. The literature largely conceptualized successful treatment as complete and sustained abstinence from drugs and alcohol and did not consider other factors related to recovery, such as those put forth in the Betty Ford Institute’s consensus panel definition [8]. This narrow definition of treatment success could be expanded in future research to include a range of patient-centered outcomes. These other outcomes could include measurements of mental health, employment, familial stability, and community service.

Lastly, on the theme of staffing and patient services, many similarities between and within countries were identified in the literature. Facilities mentioned using programming based on the Alcoholics Anonymous and Narcotics Anonymous (AA/NA) principles and emphasized that recovery is a long-term process that does not end when one is discharged from treatment [44, 54]. This treatment philosophy is consistent with the need to evaluate the role of inpatient and residential treatments in the context of an ongoing recovery process, where a single treatment episode cannot be viewed as a success or failure solely on the achievement of complete remission from SUD. While different models of treatment were mentioned, the Minnesota Model appeared to be the most commonly used [44, 54]. Many facilities employed clinical staff, most commonly nurses and physicians, in addition to mental health and addiction counselors, social workers, and administrative staff [24, 37, 55]. Significantly, studies included in the review did not report on whether those with lived SUD experience are members of the treatment teams in inpatient and residential facilities in a paid or voluntary capacity, whose inclusion in treatment teams is suggested in the WHO and UNODC guidelines [9]. This does not necessarily confirm that those with lived SUD experience or peers are not included on existing treatment teams in the study countries but offers an area for further inquiry. The initiation of people in SUD treatment into the principles of AA/NA indicates that peer support is viewed as an essential component of ongoing recovery.

Some unique service delivery mechanisms were mentioned in the literature, such as Uganda's mobile detoxification and rehabilitation unit [61]. Furthermore, there was a large variation in patients' durations of stay observed in the literature, with a duration of inpatient or residential treatment ranging from one week to several years [44, 45, 54]. Lastly, while a couple of studies indicated that medication-assisted therapy was absent from the sampled facilities [45, 53, 54], there was a limited exploration of how medications for SUD are utilized. Their absence in inpatient and residential settings could indicate that they are more frequently used in outpatient settings in SSA, or not at all.

## Limitations

Multiple limitations to this study warrant discussion. First, the publication language was restricted to English, which could have excluded studies that otherwise would meet the inclusion criteria. However, empirical studies have demonstrated that restricting systematic reviews to English does not substantially impact findings [62]. Second, included studies had to be published from the year 2000 to the time of the article search. While this could have excluded valuable information from the study, the authors decided on this timeframe to ensure that the information being reported was most likely to reflect the contemporary body of knowledge of SUD treatment in SSA.

## Conclusion

While there is a substantial volume of research regarding inpatient and residential SUD treatment in SSA, there are significant gaps in the literature. These gaps are particularly significant as they relate to exploring diverse patient-centered outcomes following residential or inpatient SUD treatment. Further research that focuses on a range of longitudinal outcomes and that does not rely solely on substance use recurrence as an indicator of treatment success would better reflect the range of psychosocial and health outcomes experienced by patients following inpatient and residential treatment. Furthermore, future research should not only involve those who are currently in treatment, but those who desire treatment but are unable to access it due to structural issues such as geographic or cost barriers, and non-structural factors including stigma and discrimination. The existing literature begins to explore barriers to treatment but could be expanded by further investigation into how these barriers can effectively be addressed to expand access. Additionally, a better understanding of the usage of medication in inpatient and residential SUD treatment settings in SSA would also strengthen the body of literature on this topic. Addressing these gaps in the literature will lead to a better understanding of how SUD treatment in SSA can better meet the WHO and UNODC standards of being available, accessible, affordable, evidence-based, and diversified.

### Supplementary Information

**Additional file 1. **

**Additional file 2. **

## Data Availability

Articles included in this scoping review were available through PubMED [((((substance use disorders) OR (drug abuse)) OR (substance dependence)) AND (treatment) AND (inpatient OR residential OR rehabilitation OR facilities)) AND (Africa OR sub-Saharan Africa)—Search Results—PubMed (nih.gov)] Scopus and African Index Medicus [ Search | Global Index Medicus (bvsalud.org)]

## Abbreviations

AA: Alcoholics Anonymous
AOD: Alcohol and Other Drugs
AU: African Union
DALY: Disability-adjusted Life Year
NA: Narcotics Anonymous
NGO: Non-governmental Organization
PRISMA-ScR: Preferred Reporting Items for Systematic Reviews and Meta-Analyses extension for Scoping Reviews
PWUD: People who use drugs
RAR: Rapid Assessment Response
SDGs: United Nations Sustainable Development Goals
SQM: Services Quality Measures
SSA: Sub-Saharan Africa
SUD: Substance Use Disorder
TC: Therapeutic Community
UNODC: United Nations Office on Drugs and Crime
WHO: World Health Organization

## Footnotes

1 These terms used to describe racial/ethnic groups in South Africa refer to demographic markers that are still used in South Africa today, but their use in this paper does not signify an endorsement of their usage by the study authors. These terms were utilized within included studies to highlight salient groupings of people related to ongoing health and socioeconomic disparities within the country.
