# Supplementary material for: Residential and inpatient treatment of substance use disorders in Sub-Saharan Africa: a scoping review
Source: Subst Abuse Treat Prev Policy. 2024 Jan 11;19:6. doi: 10.1186/s13011-023-00589-0 (PMC10782522; doi:10.1186/s13011-023-00589-0)
Supplement: Supplementary file 1 — Additional file 1. [file 13011_2023_589_MOESM1_ESM.docx]

**Additional file 1. Final Searches**

**Pub Med, April 10, 2023**

Search: **((((substance use disorders) OR (drug abuse)) OR (substance dependence)) AND (treatment) AND (inpatient OR residential OR rehabilitation OR facilities)) AND (africa OR sub-saharan Africa)**

("substance related disorders"[MeSH Terms] OR ("substance related"[All Fields] AND "disorders"[All Fields]) OR "substance related disorders"[All Fields] OR ("substance"[All Fields] AND "disorders"[All Fields]) OR "substance use disorders"[All Fields] OR ("substance related disorders"[MeSH Terms] OR ("substance related"[All Fields] AND "disorders"[All Fields]) OR "substance related disorders"[All Fields] OR ("drug"[All Fields] AND "abuse"[All Fields]) OR "drug abuse"[All Fields]) OR ("substance related disorders"[MeSH Terms] OR ("substance related"[All Fields] AND "disorders"[All Fields]) OR "substance related disorders"[All Fields] OR ("substance"[All Fields] AND "dependence"[All Fields]) OR "substance dependence"[All Fields])) AND ("therapeutics"[MeSH Terms] OR "therapeutics"[All Fields] OR "treatments"[All Fields] OR "therapy"[MeSH Subheading] OR "therapy"[All Fields] OR "treatment"[All Fields] OR "treatment s"[All Fields]) AND ("inpatient s"[All Fields] OR "inpatients"[MeSH Terms] OR "inpatients"[All Fields] OR "inpatient"[All Fields] OR ("residential"[All Fields] OR "residentially"[All Fields]) OR ("rehabilitant"[All Fields] OR "rehabilitants"[All Fields] OR "rehabilitate"[All Fields] OR "rehabilitated"[All Fields] OR "rehabilitates"[All Fields] OR "rehabilitating"[All Fields] OR "rehabilitation"[MeSH Terms] OR "rehabilitation"[All Fields] OR "rehabilitations"[All Fields] OR "rehabilitative"[All Fields] OR "rehabilitation"[MeSH Subheading] OR "rehabilitation s"[All Fields] OR "rehabilitational"[All Fields] OR "rehabilitator"[All Fields] OR "rehabilitators"[All Fields]) OR ("facilities"[All Fields] OR "facility"[All Fields] OR "facility s"[All Fields])) AND ("africa"[MeSH Terms] OR "africa"[All Fields] OR "africa s"[All Fields] OR "africas"[All Fields] OR ("africa south of the sahara"[MeSH Terms] OR ("africa"[All Fields] AND "south"[All Fields] AND "sahara"[All Fields]) OR "africa south of the sahara"[All Fields] OR ("sub"[All Fields] AND "saharan"[All Fields] AND "africa"[All Fields]) OR "sub saharan africa"[All Fields]))

**SCOPUS, April 10, 2023**

**Results: 502**

Search: (TITLE-ABS-KEY-AUTH ( ( Africa OR sub-Saharan Africa ) AND ( substance AND abuse ) AND ( treatment OR facilities OR rehabilitation OR inpatient OR residential ) ))

**African Index Medicus, April 10, 2023**

Results 118, 112 (English only)

Search: (tw:(substance abuse)) OR (tw:(substance use disorder )) OR (tw:(drug abuse)) OR (tw:(substance dependence))
