# Supplementary material for: Residential and inpatient treatment of substance use disorders in Sub-Saharan Africa: a scoping review
Source: Subst Abuse Treat Prev Policy. 2024 Jan 11;19:6. doi: 10.1186/s13011-023-00589-0 (PMC10782522; doi:10.1186/s13011-023-00589-0)
Supplement: Supplementary file 2 — Additional file 2. [file 13011_2023_589_MOESM2_ESM.docx]

| **Additional file 2** |  |  |  |
| --- | --- | --- | --- |
| **Title** | **Author(s) and Study Year** | **Study Country** | **Key Findings** |
| *Assessing the revised Clinical Institute Withdrawal for Alcohol Scale use at Stikland Hospital* | Mudappah C, Weich L, 2023 | South Africa | - Retrospective cohort study of 135 patients testing pre- and post- implementation of Revised Clinical Institute Withdrawal for Alcohol Scale (CIWA-Ar) on benzodiazepine prescription during alcohol withdrawal at Stikland Psychiatric Hospital - 2017 group (post-implementation) in the unit had a lower percentage of patients that required benzodiazepines (33.8% vs. 51.4%) and a lower median total amount of benzodiazepines used during alcohol withdrawal (0 mg vs. 5 mg) - CIWA-Ar scale was effective at limiting usage of benzodiazepines in alcohol withdrawal |
| *Intravenous ketamine for severe alcohol use disorder at Moi Teaching & Referral Hospital, Kenya: a case report* | Jaguga F *et al.*, 2023 | Kenya | - Case study of 39-year-old male with alcohol use disorder (AUD), who had received treatment 6 times previously, receiving inpatient treatment at Moi Hospital was first documented patient to receive Ketamine to assist in AUD treatment - No adverse events were associated with the usage of Ketamine - The attempt was ultimately unsuccessful as the patient relapsed one-week post-treatment |
| *The role of spirituality and religiosity in psychoactive substance use among adolescents in a Nigerian Hospital* | Falade J *et al.*, 2022 | Nigeria | - Cross-sectional descriptive survey comparing religiosity of 770 adolescents (aged 14-19) admitted to the drug rehabilitation unit or with adolescents from outpatient department of ABUAD Hospital in Ado-Ekiti with no active or past substance abuse^[[1]](#footnote-1)^ - Religiosity and spirituality, measured separately, were both higher among adolescents who do not abuse substances (62.9% and 62.6%) compared to those who do (53% and 49.1%). - Religiosity and spirituality could act as a protective factor against substance abuse |
| *Long-term relapse prevention strategies among poly-substance users in Ghana: New insights for clinical practice* | Appiah R, 2022 | Ghana | - Descriptive phenomenology study of relapse prevention strategies among 14 adults (18-65) one year after discharge from psychiatric rehabilitation unit for polysubstance use - Findings suggest importance of identifying skills that promote purposeful living, incorporation of religious practice in recovery, and strengthening family support and education to family members on addiction - Opportunity to leverage collectivist Ghanaian cultural values to encourage community support of those in recovery |
| *The effect of the COVID-19 lockdown on mental health care use in South Africa: an interrupted time-series analysis* | Wettstein A *et al*., 2022 | South Africa | - Interrupted time-series analysis using 710,367 insurance claims between 2017 and 2020 exploring impact of COVID-19 lockdown on outpatient and hospital admission visits for mental health conditions, including substance use disorders - Hospital admissions rates decreased substantially after the imposition of the COVID-19 lockdown for all mental health conditions (OR 0.38; 95% confidence interval (CI) 0.33–0.44) and did not recover to pre-lockdown levels until 1 June 2020. - Admission for alcohol withdrawal increased after the lockdown (OR 1.36;95% CI 0.46-4.2) likely in part due to the ban on alcohol sales |
| *COVID 19—impact on substance use treatment utilization and provision in South Africa* | Harker N *et al.*, 2022 | South Africa | - Cross-sectional study of 63 substance use disorder treatment facilities (39 residential) using South African Community Epidemiology Network on Drug Use (SACENDU) data to assess impact of COVID-19 on treatment admissions, as well as data from an online survey of SUD treatment providers - 30 providers (47.6%) believed treatment need for SUD had remained constant during COVID-19, yet 32 (50.7%) believed that treatment was less available during the pandemic - 20 facilities (31.7%) reported staying fully operational during the lockdowns, 7 (11%) closed, and 36 (57.1%) limited-service offerings |
| *A descriptive survey of substance use treatment facilities in Uasin Gishu County, Kenya* | Jaguga F *et al.*, 2022 | Kenya | - Cross-sectional survey of substance use treatment facility capacity in Uasin Gishu County found 6 facilities (1 government-run and 5 private) comprising 174 beds (for population of 1.1 million) of which only 33 (19%) were allocated to females and 0 for children or adolescents - One facility accepted insurance, with out-of-pocket payment being the most common form of payment for treatment fees which ranged from $700-$2000 for a 90-day stay - There were 63 health care professionals across facilities and all facilities had at least one licensed addiction counselor and one psychologist, 3 (50%) of facilities employed at least one nurse, and 4 (67%) had a psychiatrist in residence or on-call |
| *Drugs of Abuse among In-Patients Receiving Treatment for Substance Use Disorders in a Tertiary Health Care Center in South-South Nigeria: An Exploratory Qualitative Study* | Okafor CJ *et al.,* 2022 | Nigeria | - Exploratory qualitative study through of 15 adult males receiving inpatient drug treatment at Federal Neuropsychiatric Hospital of Calabar of which mean sample age was 33, 14 (93%) were unmarried, and 5 (33%) were unemployed - Most respondents reported their first substances used were either alcohol, cannabis, or tobacco - While discussing prescription drugs, respondents reported usage of tramadol, codeine, flunitrazepam, as common substances of abuse and less common means of getting high mentioned included inhalation of soak away fumes, formaldehyde, and gammalin (pesticide) |
| *Drugs and alcohol Use patterns among those seeking care in urban rehabilitation centres before and during early months of COVID-19 in Uganda* | Tumwesigye N M *et al.,* 2022 | Uganda | - Cross-sectional study of 10 SUD treatment centers (n=754) with residential offerings and established record-keeping system found patient population with median age of 32, comprised of mostly men (85%), generally well-educated (84% finished secondary school) - The most common substances of abuse were alcohol (52%), cannabis (19%), cocaine (13%), and opioids (8%) - Of the 291 who had been treated for SUD before, 66% reported that family or friends paid for their treatment, 17% from the government, and 13% from personal savings - Majority of study participants (72%) reported spending between $2.70 and $27 per week on drugs or alcohol |
| *Factors associated with patient-reported experiences and outcomes of substance use disorder treatment in Cape Town, South Africa* | Myers B *et al.*, 2022 | South Africa | - Quantitative analysis of secondary data from 1097 patient records and survey data found that White and Coloured patients were less likely to perceive difficulty than Black African patients (aOR = 0.23; 95% CI 0.11–0.51; aOR = 0.28; 95% CI 0.11–0.71) - Patients who reported a comorbid mental illness were also more likely to report suboptimal treatment than those without a comorbid mental illness ( aOR = 1.91; 95% CI 1.16–3.14, as well as those in residential treatment compared to outpatient treatment (aOR = 1.69; 95% CI 1.08–2,64) - The odds of perceiving sub-optimal treatment also increased as years of substance use increased (aOR=1.05; 95% CI 1.00-1.05) - Women were less likely than men to report suboptimal outcomes (aOR=.57; 95% CI 0.35-0.93), and patients who did not complete their treatment were three times as likely to report suboptimal outcomes (OR=3.03; 95% CI 1.33-6.86) |
| *Treating alcohol use disorder in the absence of specialized services - evaluation of the moving inpatient Treatment Camp approach in Uganda* | Ertl V *et al*.,2021 | Uganda | - Uncontrolled treatment study piloting mobile week-long mixed-gender inpatient treatment intervention for AUD in Uganda with 25 initial participants (23 male, 2 female, median age=40.6) including initial medical examination, detoxification, addiction-related psychoeducation, and introductory Alcoholics Anonymous (AA) meetings - 23 participants (92%) had relapsed at one year, but Alcohol Use Disorder Identification Test (AUDIT) scores decreased among all participants at one year follow-up with none of the participants had AUDIT scores indicating dependency - Mean audits scores fell from 27.92 pre-intervention to 6.79 at one-year follow-up - Total cost of the intervention was $111 per participant for one week of treatment |
| *Substitute Behaviors following Residential Substance Use Treatment in the Western Cape, South Africa* | Sinclair D *et al.,*2021 | South Africa | - Longitudinal cohort study of 137 adults (87 men, 50 women) discharged from residential SUD treatment facility in Western Cape, South Africa from 4–12-week treatment program - At follow-up (ranging from 63-294 days post-discharge) 50 cases (36.5%) were found to be engaging in substitute addictive behaviors, 55 (40.1%) abstained, and 32 (23.4%) had restarted substance use - Of the 50 reporting substitute addictive behaviors, 21 reported multiple. Most common were love (e.g., thoughts, feelings, behaviors about love and relationships) (24), caffeine (11), overeating (9), exercise (8), cigarettes (8), social media (7), religion (7) |
| *A Profile of Substance Abuse Clients Admitted to an Inpatient Treatment Centre in Tshwane, South Africa* | Mokwena K, Shandukani F, Fernandes L,  2021 | South Africa | - Cross sectional study of 215 adults (173 male, 42 female, mean age=27 years) admitted to inpatient SUD treatment facility in Pretoria found the substance most leading to admission was Nyaope (65.6%), followed by marijuana (29.3%), CAT (18.6%), crystal meth (14.9%), alcohol (13%), mandrax (11.6%) - 90 participants (42%) reported receiving previous treatment, citing peer pressure, unemployment, family problems, and lack of willpower as the reasons for relapse - Average age of first substance use ranged from 8-43 (mean=17) - Racially, clients were Black African (82%), Coloured (15%), Whites (2%) and Indian (1%) and unemployment was high (77%) |
| *Correlates of Self-Reported HIV Testing Among Patients in Specialized Substance Abuse Treatment Centers in South Africa* | Cummings B *et al.*, 2021 | South Africa | - Secondary data analysis of 87,339 patient admissions using SACENDU data from 86 treatment sites between 2012 and 2017 of which 31.4% were inpatient treatment admissions - Patients whose primary substance was alcohol, methamphetamine, or methaqualone, all had a higher likelihood of HIV testing prior to admission - Children and adolescents were found to be less likely to have tested for HIV (OR=0.33 95% CI: 0.29–0.38, and OR=0.59, 95% CI: 0.52–0.67), with those in the 30-39 age range reporting highest likelihood of prior HIV testing (OR=2.29, 95% CI: 2.02–2.59) - Women were more likely to have tested than men (OR=1.51, 95% CI: 1.46–1.56) and clients without an education were more likely to have tested than more educated clients (OR=1.13, 95% CI: 1.05–1.21) |
| *A qualitative exploration of the role of NGOs in the recovery support for persons with substance use disorders in a low-income African setting* | Asante KO, Asiama-Sampong E, Appiah R, 2021 | South Africa | - Descriptive qualitative study with 8 SUD administrators or senior practitioners working in NGOs based in Southern Ghana found that most NGOs offering SUD services are also providing housing for clients in treatment, spanning from a couple of weeks to several months - NGOs coordinate provision of recovery services, including the employment of resident and non-resident clinicians (psychiatrists and psychologists), medication management, and detoxification services - Staff working in the NGOs providing these services are largely dependent on foreign aid and donations, and could benefit from more governmental support |
| *Predictors of controlled prescription drug non-medical and lifetime use among patients accessing public mental health services in Uganda: a cross-sectional study* | Kamba PF *et al.*,2021 | Uganda | - Cross-sectional survey of 1275 patients from three psychiatric facilities in Uganda found non-medical usage of controlled prescription drugs (CPD) among 16.8% of the 988 who underwent urine testing, with benzodiazepines most common and 1.2% were found positive for other illicit drugs - Those receiving inpatient treatment were more likely to engage in non-medical use of CPD (OR=10.90, p<0.001) and tobacco consumption was also associated with non-medical CPD usage (OR=1.85, p=0.041) - CPD usage is even more common than other illicit drug usage, and should be screened for routinely particularly among high-risk groups |
| *Factors associated with the successful completion of a substance rehabilitation programme at a psychiatric training hospital* | Dreyer J *et al.*,2020 | South Africa | - Retrospective cross-sectional study using 2013-2014 patient data from 119 admissions to the Substance Rehabilitation Unit (SRU) at Weskoppies Hospital found that 90 (76%) completed and 29 (24%) did not complete the 6-week program - Reasons for non-completion included disobeying unit rules (n = 19), including substance use during admission (n = 9), lack of continued motivation (n = 7) and abscondment (n = 4). - Patients with higher levels of education and those who had never received a disability grant were statistically more likely to complete rehabilitation (p=.004) - Non-completers had higher rates of substance-induced disorders than completers (41% vs. 30%) and higher rates of Nyaope usage (34.5% vs. 9%) |
| *An Inverse Relationship Between Alcohol and Heroin Use in Heroin Users Post Detoxification* | Morgan N, Daniels W, Subramaney U, 2020 | South Africa | - Longitudinal study of 300 heroin users admitted to inpatient SUD treatment were evaluated upon entry and at 3- and 9-months post-discharge found that upon entry 86.3% had used marijuana, 26% crack cocaine, 19.3% crystal methamphetamine and 18.3% methaqualone in the past month in addition to heroin - At 3-month follow-up, 65.5% had continued to use heroin, and alcohol usage among those discharged had increased from 16.3% of clients upon entry to 55.2% at 3-month follow-up and 45.8% at 9-month follow-up - Only 11.6% of clients had received any community-based SUD services at 9-month follow-up, mostly peer-support meetings |
| *Clinical characteristics and treatment outcomes of women with heroin dependence in Johannesburg, South Africa* | Morgan N, Daniels W, Subramaney U, 2020 | South Africa | - Longitudinal study of 44 female heroin users evaluated upon entry into inpatient SUD treatment facility and at 3- and 9-month post-discharge found that upon entry 40% of women were HIV positive, 50% had engaged in sex work, and 27% were injection heroin users, and 75% had been diagnosed with a comorbid mental illness - 37 (84%) and 30 (68%) were reached for follow-up at 3- and 9-month post-program completion of which only 6 were abstinent from all substances at 3 months and 2 at 9 months - When comparing averages among male and female heroin users evaluated at treatment entry, women had a higher prevalence of HIV infection and mental illness |
| *Drug Treatment Presentations at a Treatment Centre in Southern Nigeria (2015–2018): Findings and Implications for Policy and Practice* | James BO *et al.*, 2019 | Nigeria | - Retrospective case review of 212 patient records from Drug Abuse Treatment, Education, and Research Unit at the Federal Neuropsychiatric Hospital between 2015 and 2018 found that 93.4% of patients treated were male, with 49.5% of clients self-reporting first substance use between 10 and 19 years of age, and 58.8% unemployment - The primary substance leading to treatment was cannabis (58%), alcohol (21.3%), and opiates (15.6%) - 53.7% of patients had a co-occurring disorder, mostly commonly mental disorders (76.3%), followed by cardiovascular diseases (7.8%) and respiratory disease (5.3%) - The vast majority had previously tested for HIV (88.6%), but few had received Hepatitis C testing (15.5%) |
| *South African service users’ perceptions of patient‐reported outcome and experience measures for adolescent substance use treatment: A qualitative study* | Myers B *et al.*, 2019 | South Africa | - Focus group discussions (FGDs) with 38 adolescents receiving inpatient or outpatient SUD treatment for adaptation of South African Addiction Treatment Services Assessment (SAATSA) for usage with adolescents found achieving sobriety was primary indicator of success noted in FGDs, but also improved quality of life, ability to set and achieve goals, and improved relationships - Participants noted building motivation for change, problem-focused and emotional coping skills, recreational alternatives to drug use, and post-treatment services as key therapeutic needs - Participants also noted importance of age-appropriate care, trustworthy staff, confidentiality, and shared decision-making between patients and providers |
| *A prospective observational study of heroin users in Johannesburg, South Africa: Assessing psychiatric comorbidities and treatment outcomes* | Morgan N, Daniels W, Subramaney U, 2019 | South Africa | - Longitudinal study of 300 heroin users upon entry to treatment facility and at 3- and 9-months post discharge found that at 3-month follow-up only 6.3% were abstinent from all substance and 65.5% had continued heroin use - At both follow-up periods, while heroin usage declined, there were increased rates of alcohol use, crystal methamphetamine use, and instances of major depression (p<.0001) - The majority (53%) of clients did not know their HIV status and none of the clients were receiving medication-assisted therapy (MAT) post-discharge |
| *A Mixed-Methods Evaluation of the Implementation of a Performance Measurement System for South Africa’s Substance Use Treatment Services* | Myers B *et al.*, 2019 | South Africa | - Mixed-methods evaluation surveyed 81 SUD service providers and conducted in-depth interviews with 26 SUD service providers about their facility’s implementation of the Service Quality Measurement (SQM) reporting requirements - Participants identified perceived utility of the system in including treatment quality, compatibility of the reporting system, and simplicity as the primary facilitators of implementation - Participants stated that the SQM was easy to implement, though some noted difficulties in interpreting feedback and incorporating it into service provision modifications - Some participants noted transitioning to an electronic record-keeping system would also ease administrative burden |
| *Dual Diagnosis: How Adults Diagnosed with Bipolar Disorder Experience Inpatient Substance Abuse Treatment for Stimulant Use Disorder* | Van Zyl K, Geyer S, 2019 | South Africa | - Exploratory qualitative study with 4 participants in inpatient SUD treatment with a dual diagnosis of bipolar disorder (BD) and stimulant use disorder found participants reported stimulant use a means of coping with mood changes associated with BD - Participants noted that despite being medicated for BD they still experienced frequent mood changes that triggered cravings for stimulant usage, which they often substituted with food, sugar, caffeine, cigarettes, or self-mutilation - Participants noted the importance of exercise in their recovery, but noted that the psychological counseling offered to them was not frequent enough and they felt they could benefit from twice weekly therapy sessions |
| *Psychiatric comorbidity among alcohol-dependent individuals seeking treatment at the Alcohol Rehabilitation Unit, Stikland Hospital* | Gabriels CM, Machari M, Weich L, 2019 | South Africa | - Cross-sectional study of 101 adults (64.5% male, 35.5% female) admitted to Alcohol Rehabilitation Unit over 6-month period found 62.4% also had a co-occurring psychiatric disorder - The most common of these disorders was anxiety disorders (42.6%) which includes agoraphobia, social phobia, and generalized anxiety and major depressive disorder (29.7%) - 13% had a comorbid substance use disorder other than alcohol use disorder (AUD) |
| *An Exploration of Aftercare Services for Female Drug Users* | Mhangwa T, Kasiram M, Zibane S, 2018 | South Africa | - Qualitative study with 6 female drug users who attended either inpatient or outpatient SUD treatment and 5 service providers working in a treatment facility found need for more aftercare services following treatment using biopsychosocial-spiritual model - Service providers noted biological component of addiction, particularly its role in cravings, as well as past trauma of many women with SUD including rape, childhood trauma, abortion, and abuse - Patients noted need for half-way house after treatment, but noted difficulty of finding a location without drugs available, as well as familial relationship challenges after treatment - Study concluded need for more research into cravings, lack of male support, and potential for more government funding to SUD treatment services |
| *Illicit drug use and violence in acute psychosis among acute adult admissions at a South African psychiatric hospital* | Witcomb R *et al.*, 2018 | South Africa | - Descriptive audit of 535 patient records from psychiatric hospital (65.6% male, 34.4% female) of patients admitted for acute psychosis found that 49% admitted patients used an illicit substance (63% of males, and 24% of females) - Majority of substance users were between 18-35 across both genders (83.1% male substance users, 73.8% female substance users) - Only 5.5% of those in sample had utilized formal rehabilitation services |
| *Substance abuse treatment engagement, completion and short-term outcomes in the Western Cape province, South Africa: Findings from the Service Quality Measures Initiative* | Myers B *et al.*, 2018 | South Africa | - Quantitative analysis of secondary data from Service Quality Measurement (SQM) data of 1,094 treatment episodes (inpatient and outpatient) found residential treatment lasted between 4 and 16 weeks *(*M*= 8.26;*SD*= 3.72)* - 94% of patients in residential setting completed their treatment compared to 48% in outpatient programs, and 92% of residential patients were abstinent at treatment end compared to 34% in outpatient setting - Being older than 25, reporting heroin use, daily drug use compared to less frequent usage, engagement in treatment, obtaining residential treatment, and shorter program duration were associated with greater odds of treatment completion |
| *Adverse childhood experiences among patients with substance use disorders at a referral psychiatric hospital in Kenya* | Kiburi SK *et al.*, 2018 | Kenya | - Descriptive cross-sectional study of 134 adults admitted to inpatient rehabilitation for SUD (88.1% male, 11.9% female) found 43.3% of participants had a family history of SUD - 82.1% reported alcohol usage and 93% had experienced at least one Adverse Childhood Experience (ACE), with the most common reported growing up with one or no parents in their household - Emotional abuse was significantly associated with tobacco use (A.O.R = 5.3 (1.2–23.9)) and sedative use (A.O.R = 4.1 (1.2–14.2)). - Childhood exposure to physical abuse was associated with cannabis use [A.O.R = 2.9 (1.0–7.90)] |
| *Alcohol and other drug use among women seeking substance abuse treatment in the Western Cape, South Africa* | Dada S *et al.*, 2018 | South Africa | - Quantitative secondary data analysis using SACENDU data from 51 AOD treatment centers, representing 74,368 patient visits of which 16,656 (22.4%) were female patients with a mean age of 29.5 years old (range 10-85) - Racially, 63.3% of women were Coloured, followed by 28.3% White, and 6.3% Black African - 50.5% were admitted as outpatients vs. 49.5% as inpatients and 26% reported receiving prior AOD treatment - Between 2000 and 2009 there was a slight increase in proportion of alcohol-related treatment admissions for women (34.1% vs. 37.3%) and heroin saw a rapid growth between 2000 and 2013 (4.8% vs. 5.6%) - Findings show that while fewer women than men are accessing AOD treatment, the proportion of women is increasing in the Western Cape province |
| *Prognostic Value of Motor Timing in Treatment Outcome in Patients With Alcohol- and/or Cocaine Use Disorder in a Rehabilitation Program* | Young SY *et al.*, 2018 | South Africa | - Prospective study of 74 adult inpatients being treated for alcohol and/or cocaine use disorder hypothesized that motor coordination, planning abilities, synchronization, and decision-making abilities would have prognostic values in treatment outcomes - The only test found to have prognostic value was the Spatial Tapping Test that explained 27% of self-efficacy among alcohol users and 25% self-efficacy among cocaine users at discharge |
| *Openness to Adopting Evidence-Based Practice in Public Substance Use Treatment in South Africa Using Task Shifting: Caseload Size Matters* | Magidson JF *et al.*, 2018 | South Africa | - Mixed-methods study with staff from 11 SUD treatment centers (1 residential, all with outpatient services) found that staff with smaller caseloads were more open to incorporating evidence-based practices (EBP) into their work - In this sample, 24% of staff had over 40 clients on their caseload - Staff members who were already using cognitive behavioral therapy (CBT) were more open to EBP than those who were not |
| *Class-Based Chronicities of Suffering and Seeking Help: Comparing Addiction Treatment Programs in Uganda* | Vorholter J, 2017 | Uganda | - Ethnographic fieldwork including qualitative research with 35 psychology and psychiatry SUD service providers in Kampala and Gulu found major differences in residential SUD treatment between private facilities, and smaller less-formalized donor-funded treatment venues - One private treatment facility was found to charge about $20 per day, with residents staying between 3-6 months, many of whom were from wealthy backgrounds, had lived or traveled abroad, and were familiar with Western treatment models for SUD - Sobriety Support private treatment center reported 65% treatment success in 2012 among participants one-year post discharge, and used Minnesota Model to design 3-month treatment program - Private facilities contrasted with non-profit CASAT mobile tent treatment venue |
| *Identifying factors associated with the discharge of male State patients from Weskoppies Hospital* | Prinsloo RG, Swanepoel A, Lippi G, 2017 | South Africa | - Cross-sectional study of 128 male patients currently in psychiatric care (n=68) and those who have been discharged (n=60) found that a lack of substance use during admission (p= 0.027), an absence of a diagnosis of substance use disorder (p= 0.013) was associated with higher likelihood of discharge - Within the population of admitted patients, 77.9% reported substance use and 2.9% reported substance dependence - Within the discharged group, 58.3% reported substance use and 1.7% reported substance dependence |
| *Readiness to adopt a performance measurement system for substance abuse treatment: Findings from the Service Quality Measures initiative* | Myers B *et al.*, 2017 | South Africa | - Cross-sectional study of 81 SUD service providers across 13 publicly funded treatment sites found high levels of readiness to adopt the Service Quality Measurements (SQM) initiative - In multi-variate analysis, awareness and leadership support were correlated positively with readiness to adopt to SQM - In bivariate analysis, caseload size was positively correlated with readiness to adopt which is likely the result of providers with larger caseloads belief that SQM system could reduce administrative burden |
| *Providers’ perceptions of the implementation of a performance measurement system for substance abuse treatment: A process evaluation of the Service Quality Measures initiative* | Myers B *et al.*, 2016 | South Africa | - Qualitative study with 15 SUD service providers across 6 treatment sites (inpatient and outpatient) found all but one provider believed the Service Quality Measures (SQM) initiative was feasible to implement - Participants believed SQM initiative could help improve quality of services, but some noted that the timing of implementation posed a challenge that led to refusal by some treatment center staff - The ability to read and complete the South African Addiction Treatment Services Assessment (SAATSA) was a barrier to client participation - Participants generally felt the implementation protocols were easily understandable, with the exception of a lack of clarity on when to complete certain components |
| *"Not on the agenda": A qualitative study of influences on health services use among poor young women who use drugs in Cape Town, South Africa* | Myers B, Carney T, Wechsberg WM, 2016 | South Africa | - Qualitative study with 23 women who use drugs (age 16-21) conducted through focus group discussions and 14 in-depth interviews with SUD service providers found poor young women who use drugs are often excluded from health policy planning, which poses a barrier to their access of SUD services - Service providers acknowledged that young women who use drugs often have complex medical and social problems that go largely unaddressed - Women who use drugs reported stigma surrounding women using drugs, contrasted with a less discriminatory attitude towards men who use drugs, which lessens their motivation to seek treatment |
| *Substance use and duration of untreated psychosis in Kwa-Zulu Natal* | Davis GP *et al.*, 2016 | South Africa | - Cross-sectional survey of 87 patients (56.3% vs. 43.7% female) receiving care at a psychiatric hospital (27 outpatient, 60 inpatient) for a severe mental illness found that life lifetime use was highest for alcohol (81.6%), tobacco (75.6%), and cannabis (49.4%) among sample population - Problematic substance use, measured through ASSIST assessment, found that for alcohol, 32.4% were at moderate risk and 5.2% were at high risk for SUD - Among cannabis users, 39.5% were at moderate risk and 6.9% at high risks - Duration of untreated psychosis (DUP) is associated with poorer treatment outcomes, and it was found that methamphetamine and methaqualone users were more likely to have longer periods of DUP |
| *Development and psychometric validation of a novel patient survey to assess perceived quality of substance abuse treatment in South Africa* | Myers B *et al.*, 2015 | South Africa | - Cross-sectional survey of 364 adults receiving SUD treatment (inpatient and outpatient) found that the South African Addiction Treatment Services Assessment (SAATSA) provided a novel way of measuring both quality and outcomes among those receiving SUD treatment - Most participants reported SAATSA was easy to use (94%) - SAATSA was revised based on findings of two pilot-studies to increased internal reliability and construct validity |
| *Addiction and treatment experiences among active methamphetamine users recruited from a township community in Cape Town, South Africa: A mixed-methods study* | Meade CS *et al.*, 2015 | South Africa | - Mixed-methods study of 360 adult methamphetamine users (56% male, 44% female) found that participants had used methamphetamine for an average of 7.06 years, with 60% of participants reporting daily use - Only 10% of participants had ever received SUD treatment, while 90% reported a desire to receive treatment - During in-depth interviews with 30 participants, barriers reported include the belief that treatment was not available to them (18%), does not work (16%), was cost prohibitive (13%), or felt shame/fear (11%) |
| *Navigating the poverty of heroin addiction treatment and recovery opportunity in Kenya: Access work, self-care, and rationed expectations* | Rhodes T *et al.*, 2015 | Kenya | - Qualitative case study with 109 adults who reported injection drug use within last 30 days (70% male, 30% female) in Nairobi, Malindi, and Ukunda - Participants reported strong desire for change but unaffordable rehabilitation centers, averaging $114 per month - Participants emphasized work done to access rehabilitative services often outweighed the actual effort they could put into personal recovery - 32 participants (29%) reported experience of residential treatment and 21 (28%) reported themselves HIV positive |
| *Codeine misuse and dependence in South Africa – learning from substance abuse treatment admissions* | Dada S *et al.*, 2015 | South Africa | - Qualitative analysis of secondary data from SACENDU in 2014 comprising 17,260 patient admissions (inpatient and outpatient) found that 435 (2.5%) treatment admissions reported codeine as a substance of abuse - Codeine was the primary substance of only 137 (0.8%) patients - Low treatment rates of people using codeine are likely due to perception that codeine is not a serious drug addiction, or can be successfully treated at the primary care level |
| *A qualitative study of referring agents’ perceptions of access barriers to inpatient substance abuse treatment centres in the Western Cape* | Isobell D, Kamaloodien K, Savahl S, 2015 | South Africa | - Qualitative study with 6 referring agents from outpatient facilities who refer SUD patient to inpatient services found that the absence of a structured referral pathway as a barrier to utilization of inpatient SUD rehabilitation - Other barriers noted included: the need for formal referrals by a social workers, mandatory detoxification and mental health services prior to inpatient admission, and extensive waiting time - One individual barrier noted was the exclusion of certain subgroups by inpatient facilities (homeless, pregnant, having a disability) |
| *Modelling the trends of inpatient and outpatient rehabilitation for methamphetamine in the Western Cape province of South Africa* | Mushanyu J, Nyabadza F, Stewart AGR, 2015 | South Africa | - Quantitative modelling using patient data from inpatient and outpatient SUD treatment facilities in Cape Town predicted decrease of inpatient admission between 2015-2020, with an increase in outpatient admissions - Projected proportion of inpatient admissions of total drug treatments in 2018 was 31% - Also projected proportion of inpatient methamphetamine inpatient to decrease, while outpatient settings would serve a great proportion of this subpopulation |
| *The association between psychopathology and substance use: adolescent and young adult substance users in inpatient treatment in Cape Town, South Africa* | Saban A *et al.*, 2014 | South Africa | - Cross-sectional study of 95 adolescent and young adult (aged 17-30) patients receiving inpatient SUD in 3 private facilities in Cape Town found a population that was predominately male (89.5%), coloured (88.4%), and Muslim (68.4%) with mean age of first substance use at 14.7 years old - Common first substances of use were cannabis (51.6%) and methamphetamine (17.9%) - Heroin (53.7%) and methamphetamine (33.7%) were the most common substances for which treatment was being sought, and cigarettes were used daily by 97.8% of the sample - 96% of the sample had some form of diagnosed psychopathology other than SUD, most common of which were anti-social personality disorder (87.4%) and conduct disorder (67.4%) |
| *Treatment for substance abuse in the 21st century: A South African perspective* | Jeewa A, Kasiram M, 2014 | South Africa | - Exploratory qualitative research involving 3 SUD treatment centers each with different treatment model: Minnesota Model, Therapeutic Community Model (TCM), and NARCONON Model - Focus group discussions with patients and structured interviews with service providers showed differences in staffing with the Minnesota Model facility included two graduates with degrees in social work and psychology, as well as a paraprofessional/life skills educator - The TCM facility had only one paraprofessional, which appeared to indicate a staff shortage, and the NARCONON model was staffed partially by former clients - Overall, facilities indicated desire to move away from “disease model” of treatment, employed multidisciplinary teams, and stated desire to utilize alternative therapies including massage, sauna, dietary interventions, and physical activity |
| *Perceived need for substance use treatment among young women from disadvantaged communities in Cape Town, South Africa* | Myers B *et al.*, 2014 | South Africa | - Secondary data analysis of 720 women who use drugs who were recruited as part of field experiment exploring factors associated with SUD treatment demand found only 46% of participants reported needing treatment - Of those 46%, 92.4% reported readiness to go to treatment - There was no statistically significant treatment demand among those testing positive or negative for methamphetamines and only 7.4% reported receiving prior treatment for SUD - Only 51.1% knew where they could go for treatment |
| *Identifying perceived barriers to monitoring service quality among substance abuse treatment providers in South Africa* | Myers B *et al.*, 2014 | South Africa | - Focus group discussions with 21 SUD treatment providers across 12 sites found lack of technology limits monitoring data collection and storage - Providers also noted that there were instances of underutilization of data collection, duplication of data collection efforts, and that service quality data collection forms needed to be improved - Providers also noted the high patient caseload, particularly in outpatient settings, but unanimously supported enhanced service quality projects |
| *Monitoring the prevalence of methamphetamine-related presentations at psychiatric hospitals in Cape Town, South Africa* | Plüddemann A *et al.*, 2013 | South Africa | - Secondary data analysis from 235 treatment admissions to psychiatric hospital showed burden that admissions related to methamphetamine (MA) are posing to psychiatric treatment facilities - MA-related admissions pose a significant burden to staff due to aggressive behavior and severe psychotic symptoms - Management and containment of MA using patients can overburden staff without capacity for more individualized attention |
| *Service Use, Charge, and Access to Mental Healthcare in a Private Kenyan Inpatient Setting: The Effects of Insurance* | de Menil VP *et al.*, 2013 | Kenya | - Cross-sectional study from Chiromo psychiatric hospital analyzed data from 455 patients over year-long period found that 31.6% of patient were admitted for SUD - Insurance paid in full for 28.8% of patients and the mean length of stay was 11.8 days - 22% of patient population were readmitted within 12 months - Patients with private health insurance stayed 36% on average compared to those with public health insurance of no health insurance |
| *Substance Abuse Inpatients' Experience of Animal Assisted Therapy* | Coetzee N, Beukes JT, Lynch I, 2013 | South Africa | - Focus group discussion with 4 residents of inpatient SUD facility found animal-assisted therapy (AAT) through a trip to a local animal park encouraged self-reflection and goal setting - AAT also was shown to be a catalyst for social interaction among residents, namely through reflected on the shared experience and sharing past experiences with animals - Social interaction was particularly important given participants limited social network during treatment |
| *Decline in adolescent treatment admissions for methamphetamine use in Cape Town* | Plüddemann A , Dada S, Parry C, 2013 | South Africa | - Quantitative analysis of secondary patient data from select time periods between 2006-2011 found that the proportion of adolescents (20 years and younger) whose primary substance was methamphetamine seeking treatment began to decline between 2006 and 2011 (58.6% vs. 24.5%) - Between 2006 and 2011 the total number of patients seeking remained stable, the proportion of adults whose primary substance was methamphetamine increase between 2006 and 2011 (28.7% vs 41.5%) - Adolescents’ decline in methamphetamine use was replaced with a higher proportion reporting cannabis usage |
| *Short-term diagnostic stability among re-admitted psychiatric in-patients in Eldoret, Kenya* | Atwoli L *et al.*, 2012 | Kenya | - Quantitative analysis of secondary data related to diagnostic changes of 114 inpatients receiving treatment in a psychiatric hospital found 8 participants (7%) were admitted for SUD - At discharge, all but one of the 8 initially diagnosed had retained their diagnosis - Upon discharge, 14 patients had total had received an SUD diagnosis - There is strong diagnostic stability of SUD in low-income treatment setting compared to other diagnoses |
| *Social service offices as a point of entry into substance abuse treatment for poor South Africans* | Burnhams NH, Dada S, Myers B, 2012 | South Africa | - Secondary data analysis comparing standardized survey data collected from 691 recipients of social welfare and those receiving SUD treatment between 2007 and 2009 found a higher proportion of substance-using women were accessing social welfare than were represented in treatment (Chi-square = 21.92 (df = 1), p < 0.001) - Black Africans and Coloured substance-using adults also sought social welfare services at higher rates than they accessed SUD treatment (Chi-square = 5.12 (df = 1), p < 0.02) and Coloured clients (Chi-square = 68.45 (df = 1), p < 0.001) - Substance-using younger adults also accessed social welfare services at higher rates than SUD treatment (t = 6.44 (df = 16773), p < 0.001) - Findings suggest social service offices could act as entry point for those underrepresented in SUD treatment |
| *Rapid assessment response (RAR) study: drug use and health risk - Pretoria, South Africa* | Dos Santos MM, Trautmann F, Kools JP, 2011 | South Africa | - Rapid assessment response used in-depth interviews and focus group with 63 adults (49 males, 14 females) receiving residential SUD treatment and 21 service providers working in residential facilities found participants reported need for more state-sponsored residential drug treatment - Cost was cited as a barrier to residential treatment, as well as the ideology of some fundamentalist Evangelical-run treatment centers - A need for more HIV services for those in treatment was also noted by participants |
| *Diagnostic characteristics of inpatients in a Western African psychiatric hospital* | Nyan O *et al.*, 2011 | The Gambia | - Cohort study of 53 patients (47 males, 6 females) in psychiatric hospital in The Gambia found that 50% of patients in cohort were being treated for SUD - Cannabis was the most used drug among cohort (41.5%), which was either used alone or in combination with stimulants/tranquilizers - About 35% of cohort had dual diagnoses, with a dual diagnosis of SUD and a mood disorder being most common |
| *Gender differences in barriers to alcohol and other drug treatment in Cape Town, South Africa* | Myers B, Louw J, Pasche S, 2011 | South Africa | - Case control study of 434 individuals receiving SUD treatment (inpatient and outpatient) and 555 controls found few variables differed across gender in terms of determinants of likelihood of accessing SUD treatment - Awareness of services, proximity to treatment, and ability to pay for treatment were statistically significantly correlated with greater treatment utilization - While both genders have similar enabling factors to SUD treatment uptake, women are likely more impacted by financial and geographic barriers to treatment and overall have lower levels of treatment utilization |
| *A descriptive survey of types, spread and characteristics of substance abuse treatment centers in Nigeria* | Onifade PO *et al.*, 2011 | Nigeria | - Descriptive survey of 31 SUD treatment centers (16 residential, 15 outpatient) in Nigeria found capacity of residential facilities to be 566 beds and had treated 1132 patients in past year - More than half of the 31 SUD treatment had been built in the preceding 10 years - Half of the facilities surveyed did not engage in any treatment quality measurements - Of the 16 residential units, none offered medication-assisted therapy (MAT), 14 (87.5%) had treatment duration between 3 months and 2 years, 11 (68.8%) had individualized treatment plans, intake assessments were conducted in 15 (93.8%), 11 (68.8%) only used paper for patient records and had no electronic system |
| *Trends in Sociodemographic and Drug Abuse Variables in Patients with Alcohol and Drug Use Disorders in a Nigerian Treatment Facility* | Adamson TA, Onifade PO, Ogunwale A, 2010 | Nigeria | - Comparative cross-sectional study using patient data from SUD treatment unit of neuropsychiatric hospital compared patient data between 1992-1997 (n=109) and 2002-2007 (n=105) found increase in duration of stay over 90 days (75.2% vs 53.2%) and higher rates of patients with comorbid psychiatric conditions (67% vs. 38.5%) in 2002-2007 time period - Cannabis was the most used drug in 2002-2007 time period (53.5%) compared to cocaine (44%) in 1992-1997 - There were greater proportions of males in both time periods (91.7%, 90.5%) - There was also earlier onset of drug use during adolescent years (10-14 years) in 2002-2007 (32.4% vs. 19.3%) |
| *Inequitable access to substance abuse treatment services in Cape Town, South Africa* | Myers B, Louw J, Pasche S, 2010 | South Africa | - Case-control study comparing people 434 people with SUD who accessed treatment and 555 people with SUD who did not find that those who cited competing financial priorities had four-fold odds of not accessing treatment - Those who did not access treatment were also more likely to be less aware of treatment options, live further from available treatment, and to report delays when trying to access SUD treatment - Provision of food vouchers may help to mitigate some of the barriers to accessing SUD care and to expand access to people with SUD from disadvantaged communities |
| *Factors Associated with Retention in Alcohol and Other Drug Treatment Among Disadvantaged Communities in Cape Town, South Africa* | Pasche S, Myers B, Adam M, 2010 | South Africa | - Secondary data analysis using 434 patient records (inpatient and outpatient) of those who had accessed residential drug treatment in the past year found that race was the only demographic variable associated with treatment retention (defined as time spent in treatment) - Black Africans were less likely to access long-term residential treatment than their Coloured counterparts, with 59% of Coloured participants in the sample reporting residential service use compared to just 13% of Black Africans - Linguistic differences in available services and financial/geographic barriers likely contribute to this disparity - Therapeutic alliance, social support, and psychological functioning were positively correlated with treatment adherence |
| *A retrospective review of trends and clinical characteristics of methamphetamine-related acute psychiatric admissions in a South African context* | Vos PJ *et al.*, 2010 | South Africa | - A retrospective review of clinical records from psychiatric ward in 2002 (n=73) and 2006 (n=75) reflect increasing trends in reported methamphetamine (MA) use in South Africa, with no MA-related admission in 2002 time period compared to 28 (37%) in 2006 time period - There were significantly greatly numbers of adolescents admitted in 2006 than 2002 (41% vs. 23%) and among adolescent admissions 61% were MA-related - There was also a greater proportion of adult MA-users among those admitted in 2006 |
| *Correlates of substance abuse treatment completion among disadvantaged communities in Cape Town, South Africa* | Myers B, Pasche S, Adam M, 2010 | South Africa | - Cross-sectional secondary data analysis of 434 patients from low-income areas of Cape Town who received SUD treatment (61.3% outpatient, 33.6% residential, 3.9% hospital-based, 1.4% detoxification only) found of those who completed treatment 40.5% were female and 59.5% were male - The two variables most positively correlated with treatment completion were therapeutic alliance and perceptions of treatment quality - Older individuals and those who had a later onset of first drug use also were more likely to complete treatment than those younger and who initiated drug use at younger ages |
| *South African Health Care Providers' Recognition of the Link Between Alcohol and HIV in their Daily Practice: A study* | Morojele NK *et al.*, 2010 | South Africa | - Pilot survey of health care service providers, including SUD service providers, regarding recognition of link between alcohol use and HIV transmission included 56 SUD programs of which 30 (54%) were residential rehabilitation centers found 96% of SUD treatment facilities inform clients about link between HIV and alcohol in general - 95% of SUD treatment centers reported including information about alcohol’s role in HIV transmission, and 93% reported they provide clients information alcohol’s role in HIV progression |
| *Perceptions of Organizational Functioning in Substance Abuse Treatment Facilities in South Africa* | Bowles S, Louw J, Myers B, 2010 | South Africa | - Qualitative study with 44 managers and 102 counselors for SUD treatment facilities (57% inpatient facilities) used Organizational Readiness for Change (ORC) measure found that directors and staff generally had similar appraisals on the ORC scale - Directors, however, scored higher on adequacy of equipment (30.22 vs. 27.36), mutual influence (39.82 vs. 36.83) and clarity of organizational mission (39.18 vs. 36.90) - Stratified by ethnicity, White directors scored lower on Motivation to Change metric (28), compared to Black and Coloured directors (37.4, 36.3). |
| *Substance abuse, treatment needs and access among female sex workers and non-sex workers in Pretoria, South Africa* | Wechsberg WM *et al.*, 2010 | South Africa | - Cross-sectional study of 506 women who were either females sex workers (FSW) or who reported engaging in unprotected sex but were not female sex workers (non-FSW) (335 FSW, 171 non-FSW) found that less than 20% across both groups had any knowledge of alcohol or drug treatment programs - Lifetime substance use was higher among FSW across all drug types - 3% of FSW and 2% non-FSW reported trying to access SUD treatment in the past year but being unable - 77.1% of FSW expressed a desire to receive treatment |
| *Stigma, treatment beliefs, and substance abuse treatment use in historically disadvantaged communities* | Myers B, Fakier N, Louw J, 2009 | South Africa | - Qualitative study among 20 people who lived or worked among historically disadvantaged communities found that limited knowledge of SUD in HDCs contribute to doubts about treatment efficacy particularly after experiencing a relapse after a single treatment episode - Participants reported that women with SUD are often perceived more negatively than men, possibly due to negative associations of substance use with sexual availability and inability to fulfill traditional female roles - These perceptions of women with SUD likely act as a barrier to women accessing care as they fear losing their children, poor treatment by providers, or being labelled as an addict |
| *Occurrence of comorbid substance use disorders among acute psychiatric inpatients at Stikland Hospital in the Western Cape, South Africa* | Weich L, Pienaar W, 2009 | South Africa | - Descriptive prevalence study of 298 patients in psychiatric unit found comorbid SUD among 23% of patients met criteria for substance abuse and 24% met the criteria for substance dependence - 9% reported a history of past substance dependence, currently in remission - Through patient self-report or collateral history, 51% of sample participants were found to have comorbid substance abuse or substance dependence |
| *Provision of Mental Health Services in South African Substance Abuse Treatment Facilities* | Myers B, Fakie N, 2009 | South Africa | - Cross-sectional study of 45 SUD treatment facilities (inpatient and outpatient) using Treatment Services Audit Questionnaire found that most facilities (73%) offered addiction counseling, but detoxification services were less common (57%) - Psychiatric services were offered by 23% of facilities in the sample, and aftercare was offered by 73% - A significantly higher proportion of inpatient facilities than outpatient facilities offered psychiatric medications (Chi-square = 26.17; *p* < 0.000) and mental health counseling (Chi-square = 19.85; *p* < 0.003) |
| *Monitoring and Evaluation of Substance Abuse Services in South Africa: Implications for Policy and Practice* | Myers B, Burnhams NH, Fakier N, 2009 | South Africa | - Cross-sectional study of 55 SUD treatment facilities (inpatient and outpatient) 56% and 48% of treatment centers in Gauteng and KwaZulu-Natal (KZN) provinces, respectively, had never completed any form of program evaluation - 76% of facilities in Gauteng and 95% in KZN had individualized treatment plans, but only had computerized file systems at rates of 33% and 40%, respectively. - Only 36% of facilities in Gauteng and 67% in KZN did routine outcome monitoring of patients, and client satisfaction was only measured at rates of 38% and 52%, respectively - Much of the monitoring takes place on ad hoc basis |
| *The Substance Abuse Treatment Workforce of South Africa* | Sodano R *et al.*, 2009 | South Africa | - Cross-sectional study of 143 SUD counselors across 24 SUD treatment facilities found that 75% of counselors were female with 36.4% identifying themselves as White, 30.8% as Black, 18.9% as Coloured (mixed race), 12.6% as Indian or Asian, and 1.4% as Cape Malay - Educationally, 62.3% held at least an equivalent of a bachelor’s degree, and just under half (49%) were registered social workers - Work settings varied with 55% of counselors reported that they worked in an inpatient setting, followed by outpatient (28.2%), residential (11.6%), alcohol or drug education classes (2.2%), and other settings, including court ordered treatment, employee assistance programs, and pastoral services (2.9%) - Credentialing of counselors included certifications like registered counselor (14.7%), certified teacher (8.4%), registered social worker (49%), registered nurse (6.3%) and registered psychologist (2.8%), among others |
| *Clinical epidemiology in patients admitted at Mathari Psychiatric Hospital, Nairobi, Kenya* | Ndetei DM *et al.*, 2008 | Kenya | - Cross-sectional study of patients in psychiatric hospital found 34.4% were being treated for SUD (third most common reason for admission) - Most common substances used were alcohol, sedatives (usually prescription), khat, and opiates - Challenge was identified in diagnosing SUD among patients with psychotic disorders |
| *Surge in treatment admissions related to methamphetamine use in Cape Town, South Africa: implications for public health* | Pluddeman A, Myers B, Parry CDH 2008 | South Africa | - Analysis of secondary data collected through the South African Community Epidemiology Network (SACENDU) observed most rapid increase between 2004 and 2006 in methamphetamine-related SUD treatment admissions ever in national history - The proportion of SUD treatment-seekers with methamphetamine as their primary substance rose from 0.3% in the first half of 2002 (n=4/1608) to 42.3% in the second half of 2006 (n=1184/2798) - Racially, Coloured South Africans were more likely to access treatment for methamphetamine-related problems than patients from other race groups (with the proportion of Coloured patients ranging from 81% to 92%) and the median age ranged 19.7 to 22.3 between 2004 and 2006 |
| *Alcohol and drug abuse: removing structural barriers to treatment for historically disadvantaged communities in Cape Town* | Myers B, Louw J, Fakier N, 2008 | South Africa | - Exploratory qualitative study with 20 SUD services providers including treatment facility staff, social service providers, social workers, and drug action committee members identified three structural barriers to SUD treatment - Barriers included a lack of strategic plan to tackle SUD (poor infrastructure, lack of collaboration), limited allocation of resources to SUD treatment, and fragmentation in service delivery |
| *Clinical profile of acutely ill psychiatric patients admitted to a general hospital psychiatric unit* | van Rensburg ABJ, 2007 | South Africa | - Retrospective audit of patient data from psychiatric unit found 438 admissions over one-year period - 6% of admitted patients had SUD as their primary reason for admission - 40% of patients had self-reported SUD, showing its comorbidity with other psychiatric illnesses for which they sought treatment |
| *Predictors of Treatment Non-Adherence in an Inpatient Substance Abuse Rehabilitation Programme* | Beuster J, Arnott R, 2007 | South Africa | - Retrospective analysis of 85 patient records at an inpatient SUD treatment facility found that no significant differences between adherent/non-adherent groups based on supplementary medication taken during treatment - There is some evidence of an association between Axis I disorders and treatment adherence, but not statistically significant and Axis II disorders were found in similar proportions between groups - The single most reliable predictor of adherence was tendency to overuse over the counter and psychiatric medication, and those with a legal history were more likely to complete treatment |
| *Cocaine treatment admissions at three sentinel sites in South Africa (1997–2006): findings and implications for policy, practice, and research* | Parry CDH, Pluddeman A, Myers B, 2007 | South Africa | - Secondary data analysis of patient records between January 1997 and December at SUD facilities found significantly higher proportions of treatment admissions related to cocaine between 1997 and 2006 - The percentage of crack cocaine users compared to HCL cocaine has fluctuated between 50% to 85% in Gauteng province and Cape Town and 31% to 66% in Eastern Cape - The proportion of patients seeking treatment who were women ranged from 10% and 31% in Cape Town between 14% and 28% in Gauteng, and between 14% and 20% in the Eastern Cape |
| *Access to substance abuse treatment services for Black South Africans: Findings from audits of specialist treatment facilities in Cape Town and Gauteng* | Myers B, Parry CDH, 2005 | South Africa | - Cross-sectional study of 55 SUD treatment facilities (inpatient and outpatient) found outpatient facilities were significantly more likely to serve a higher proportion of black clients than state inpatient, private non-profit inpatient, or private for-profit facilities - Private for-profit facilities in Cape Town and Gauteng were significantly more likely to serve a higher proportion of white clients than state inpatient, private non-profit inpatient, or private for-profit facilities and higher proportion of outpatient facilities offered a sliding scale, increasing access - A higher proportion of outpatient than inpatient facilities provide program services in a number of languages and employ African-language speaking therapists, and conduct outreach to Black African community |
| *Indicators of substance abuse treatment demand in Cape Town, South Africa (1997-2001)* | Myers B, Parry CDH, Pluddeman A, 2004 | South Africa | - Secondary data analysis of 3000 patient records from SUD treatment facilities between 1997 and 2001 found that the proportion of patients citing alcohol as their primary substance of abuse declined from 82% to 46% - Treatment demand for cannabis increased between 1997 and 2001 5% to 12% of total demand, and went from 4% to 6% for cocaine-related admissions - The proportion of patients treated for heroin also increased from 1% to 6% in the study period |
| *Trends in adolescent alcohol and other drug use: findings from three sentinel sites in South Africa (1997–2001)* | Parry CDH *et al.*, 2004 | South Africa | - Mixed methods study of 50 SUD treatment centers (in between 1997 and 2001 found that the proportion of patients under 20 increased from 5.5% to 24.1% in Cape Town and 7% to 22% in Durban, and cannabis was the most common primary substance among adolescents - Methqualone was the second most common primary substance among adolescents in Cape Town compared to alcohol in Durban - In the time study time period, the proportion of adolescents seeking treatment for cocaine decreased from 5% to 2.9%, but the proportion of adolescents reporting heroin usage as their primary substance rose from 2.1% to 9.1% and 4.7% to 7.5% in Cape Town and Gauteng, respectively |
| *Over-the-counter and prescription medicine misuse in Cape Town — findings from specialist treatment centres* | Myers B, Siegefried N, Parry CDH, 2003 | South Africa | - Retrospective study of 9,063 patient across 23 SUD treatment centers (inpatient and outpatient) between 1998 and 2000 found over the counter (OTC) and prescription medicines were the primary substance of abuse among 2.6% of patients - Among this 2.6%, 46.4% reported abuse of benzodiazepines and 44.8% reported analgesic usage - Among benzodiazepine users, 66.7% were females and 52.3% of analgesic users were female |
| The South African Community Epidemiology Network on Drug Use (SACENDU) project, phases 1-8–cannabis and Mandrax | Bhana A *et al.*, 2002 | South Africa | - Descriptive epidemiological study of patient data from 50 SUD treatment centers between 1996 and 2000 found that cannabis, or a combination of cannabis and methaqualone/mandrax was the most common primary drugs of use - In the study period, the proportion of patient receiving treatment for cannabis or cannabis and methaqualone/mandrax ranged between 5% and 30% - The mean age of patients with cannabis as their primary drug of use ranged from 19 to 23 and between 24 and 28 for methaqualone/mandrax, with females accounting for 4-18% of patients being treated for these substances - Between 1997 and 2000, between 40-60% of patients discharged from psychiatric care self-reported cannabis use |

1. The terms “substance abuse,” “substance abuser,” or “user” appear in some of the included studies. We are mindful of the changing use of language around substance use and acknowledge that the American Psychological Association has dropped the term “abuse” from its diagnostic terminology due to its association with the stigmatization of people who use drugs. The authors have made best efforts to only use these terms when they are utilized by the study authors, and their inclusion in this chart does not indicate an endorsement of these terms. [↑](#footnote-ref-1)
